# Determination of WWOX Function in Modulating Cellular Pathways Activated by AP-2α and AP-2γ Transcription Factors in Bladder Cancer

**DOI:** 10.3390/cells11091382

**Published:** 2022-04-19

**Authors:** Damian Kołat, Żaneta Kałuzińska, Andrzej K. Bednarek, Elżbieta Płuciennik

**Affiliations:** Department of Molecular Carcinogenesis, Medical University of Lodz, 90-752 Lodz, Poland; zaneta.kaluzinska@stud.umed.lodz.pl (Ż.K.); andrzej.bednarek@umed.lodz.pl (A.K.B.); elzbieta.pluciennik@umed.lodz.pl (E.P.)

**Keywords:** WWOX, AP-2α, AP-2γ, *TFAP2A*, *TFAP2C*, target genes, bladder, cancer, functional genomics, bioinformatics

## Abstract

Following the invention of high-throughput sequencing, cancer research focused on investigating disease-related alterations, often inadvertently omitting tumor heterogeneity. This research was intended to limit the impact of heterogeneity on conclusions related to WWOX/AP-2α/AP-2γ in bladder cancer which differently influenced carcinogenesis. The study examined the signaling pathways regulated by WWOX-dependent AP-2 targets in cell lines as biological replicates using high-throughput sequencing. RT-112, HT-1376 and CAL-29 cell lines were subjected to two stable lentiviral transductions. Following CAGE-seq and differential expression analysis, the most important genes were identified and functionally annotated. Western blot was performed to validate the selected observations. The role of genes in biological processes was assessed and networks were visualized. Ultimately, principal component analysis was performed. The studied genes were found to be implicated in MAPK, Wnt, Ras, PI3K-Akt or Rap1 signaling. Data from pathways were collected, explaining the differences/similarities between phenotypes. *FGFR3*, *STAT6*, *EFNA1*, *GSK3B*, *PIK3CB* and *SOS1* were successfully validated at the protein level. Afterwards, a definitive network was built using 173 genes. Principal component analysis revealed that the various expression of these genes explains the phenotypes. In conclusion, the current study certified that the signaling pathways regulated by WWOX and AP-2α have more in common than that regulated by AP-2γ. This is because WWOX acts as an EMT inhibitor, AP-2γ as an EMT enhancer while AP-2α as a MET inducer. Therefore, the relevance of AP-2γ in targeted therapy is now more evident. Some of the differently regulated genes can find application in bladder cancer treatment.

## 1. Introduction

Following the invention of high-throughput sequencing (HTS), cancer research was directed to investigate disease-related genetic and epigenetic alterations; however, this approach often inadvertently omitted the fact of tumor heterogeneity, i.e., presence of cells having various characteristics and origin [1]. Cancer cell lines, commonly found in cancer research, demonstrate considerable cell-to-cell (within cell line) or type-to-type (between cell lines) heterogeneity that is related to various aspects such as cellular signaling [2,3]. Recently, Shen et al. confirmed that the breast cancer model is heterogeneous both within and across cell lines [4]; a similar study by Qin et al. recapitulated intertumoral diversity in cell lines representing melanoma [5].

In view of the aforementioned data, this research was designed to limit the impact of heterogeneity on our previous findings [6,7] for bladder cancer (BLCA); thus assessing the influence of investigated molecules on BLCA signaling. Namely, we have recently considered it rational to investigate the biological effect of three genes on bladder cancer, i.e., WW Domain Containing Oxidoreductase (*WWOX*), Transcription Factor AP-2 Alpha (*TFAP2A*) and Transcription Factor AP-2 Gamma (*TFAP2C*), the last two encoding Activating Enhancer-Binding Protein 2 Alpha (AP-2α) and AP-2γ, respectively. In general, the first tryptophan domain of WWOX is responsible for binding the proline-rich motif of AP-2α/γ [8] which leads to sequestration of AP-2 proteins out of the nucleus and thus reduces their transcriptional activity [9]; AP-2γ is bound by WWOX more strongly than AP-2α [8]. Cellular variants of three BLCA cell lines (RT-112, HT-1376 and CAL-29), developed to obtain specific expression of these genes, demonstrated distinct phenotypes when investigating a range of biological processes through in vitro assays [6,7]. The findings indicate that WWOX and AP-2α behave as tumor suppressors and may act synergistically while AP-2γ is an oncogene that can be guided by WWOX against tumor progression. Using a type of HTS technology, Cap Analysis Gene Expression sequencing (CAGE-seq), it is intended to analyze the same cell lines from different perspective, treating them as biological replicates to improve robustness of data and inferred conclusions; this is highly preferred in RNA-seq studies since the value of technical replicates in such research is questionable [10].

In this work, it was decided to focus on signaling pathways in order to (1) provide a mechanistic/molecular explanation of previously observed phenotypes and (2) capture the similarities in cellular signaling which could help in cancer treatment, as signaling heterogeneity contributes to therapeutic failure [3]. This is of utmost importance because one of the investigated molecules, AP-2γ, is cancer-promoting and it might be suitable for targeted therapy aimed at transcription factors [11,12,13], similar to another member of the same family—AP-2δ [14]. Moreover, comparing the pathways which are differently modulated by AP-2γ to those of AP-2α and WWOX could identify relevant molecules that might play a role in anti-cancer therapy. Dissimilarity of AP-2γ from both AP-2α and WWOX would also strengthen the up-to-date conclusions related to this thematic issue. Therefore, the aim of the current study was to determine the role of the WWOX in modulating cellular pathways activated by AP-2α and AP-2γ transcription factors in bladder cancer. The use of biological replicates will highlight the necessary similarities at the molecular level (within the cellular variant), and determine unequivocal differences in the function of WWOX, AP-2α and AP-2γ. Secondarily, it will be possible to identify the genes regulated inversely by WWOX and specific AP-2 transcription factor (TF), whose difference in expression is alleviated when these two are analyzed in combination within a single cellular variant.

## 2. Materials and Methods

### 2.1. Cell Lines, Culture Conditions and Stable Transductions

**Figure 1:**
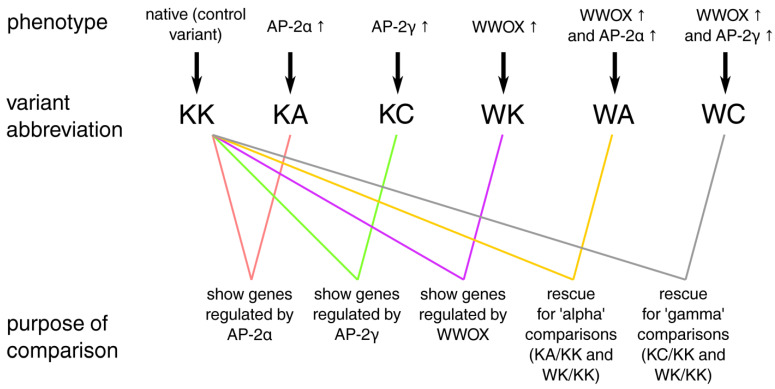
Comparisons of cellular variants and their purpose.

The cellular variants and their culture or transduction procedures are thoroughly described in previous research [6,7]. In brief, bladder cancer RT-112, HT-1376 and CAL-29 cell lines were purchased from Deutsche Sammlung von Mikroorganismen und Zellkulturen (DSMZ, Brunswick, Germany), incubated in 37 °C under 5% CO_2_ and cultured in either RPMI-1640 (RT-112) or DMEM (HT-1376, CAL-29) medium supplemented with fetal bovine serum, Antibiotic-Antimycotic and L-glutamine. Lentiviral transduction was performed via different overexpression systems for WWOX and AP-2α/γ in polybrene-containing starvation medium. Antibiotic clone selection using puromycin (WWOX) or G418 (AP-2α/γ) was preceded by exchange of viral medium to full medium. The following stable transductants were obtained for each cell line: Control/control (KK); Control/AP-2α↑ (KA); Control/AP-2γ↑ (KC); WWOX↑/control (WK); WWOX↑/AP-2α↑ (WA); WWOX↑/AP-2γ↑ (WC). In order to identify genes whose expression is inversely regulated by WWOX or AP-2 and alleviated during their combination, in this research the WA and WC variants were considered as a rescue experiment to KK: these are models where the level of WWOX/AP-2 expression is balanced to some extent compared to KA, KC or WK. Established comparisons together with their purpose are summarized in Figure 1.

### 2.2. Protein Extraction and Western Blot

The proteins were isolated using RIPA Lysis Buffer supplemented with sodium orthovanadate, phosphatase inhibitor cocktail and phenylmethylsulfonyl fluoride (Santa Cruz Biotechnology, Dallas, TX, USA), the blotting for WWOX and AP-2 TFs was then performed as previously described [6,7] to confirm successful transduction.

Western blot was also applied to validate CAGE-seq findings on the protein level. The molecule selection resulted from successive steps of the current study which are described below in Section 2.7 and Section 2.8. Anti-FGFR3 (66954-1-Ig; ProteinTech Group, Manchester UK), anti-STAT6 (51073-1-AP; ProteinTech Group, Manchester, UK), anti-EFNA1 (AMAB91423; Merck Life Sciences Sigma Aldrich, Darmstadt, Germany), anti-GSK3B (22104-1-AP; ProteinTech Group, Manchester, UK), anti-PIK3CB (21739-1-AP; ProteinTech Group, Manchester, UK), anti-SOS1 (55041-1-AP; ProteinTech Group, Manchester, UK) and anti-KIF7 (24693-1-AP; ProteinTech Group, Manchester, UK) were used as primary antibodies diluted 1:1000 (except FGFR3 antibody which was diluted 1:5000) with 1% non-fat milk in 1X TBST solution (Merck Life Sciences Sigma Aldrich, Darmstadt, Germany). Following overnight incubation, the membranes were washed three times using 1X TBST buffer and then incubated with anti-rabbit (STAT6, GSK3B, PIK3CB, SOS1, KIF7) or anti-mouse (FGFR3, EFNA1) secondary antibodies conjugated with alkaline phosphatase (Merck Life Sciences Sigma Aldrich, Darmstadt, Germany). The intensity of the bands was visualized with Novex^®^ AP Chromogenic Substrate (Invitrogen Life Technologies, Carlsbad, CA, USA). The band intensity of each protein was analyzed using ImageJ software and normalized to glyceraldehyde 3-phosphate dehydrogenase (GAPDH; sc-59540; Santa Cruz Biotechnology, Dallas, TX, USA) or β-actin (ACTB; GTX109639; GeneTex, Irvine, CA, USA). The assay was performed in biological and technical triplicates.

### 2.3. Statistical Analysis

The Shapiro–Wilk test was performed to determine the normality of distribution. The differences in relative gene expression between variants were determined by unpaired *t*-test with Welch’s correction; the results with a *p*-value < 0.05 were considered statistically significant.

### 2.4. RNA Isolation, CAGE Library Preparation, Sequencing, Mapping and Gene Expression

RNA was isolated according to the protocol provided with the Extracol reagent (EURX, Poland); total RNA quality (integrity number of at least 7.0) was evaluated using an Agilent 2100 Bioanalyzer (Agilent Technologies, Santa Clara, CA, USA). Complementary DNA (cDNA) synthesis from total RNA was achieved using random primers (CAGE library preparation Kit; K.K. DNAFORM, Yokohama, Japan). The 5′ cap-located ribose diols in RNAs were oxidized and biotinylated. Following cap-trapping, streptavidin beads enabled RNA/cDNA hybrids selection. RNA digestion using RNaseI/H was followed by linker ligation to 5′ and 3′ cDNA ends, allowing to construct double-stranded cDNA libraries.

Sequencing of CAGE libraries was performed with the use of 75 nt single-end reads on a NextSeq 500 sequencer (Illumina, San Diego, CA, USA). The raw data from the CAGE experiment were deposited in NCBI Gene Expression Omnibus (GEO) Database with accession number GSE193659. Fastq files were quality checked via FastQC tool (v0.11.9) and obtained tags were mapped to human hg38 genome using the Burrows–Wheeler Alignment tool (v0.7.17). Reads that were still unmapped were processed using Hierarchical Indexing for Spliced Alignment of Transcripts (v2.0.5). Tags were clustered using the modified Paraclu from the RECLU pipeline [15]. Regions having an overlap of 90% between replicates were extracted via BEDtools (v2.12). Clusters with Irreproducible Discovery Rate ≥0.1 and >200 bp were rejected.

### 2.5. Identification of Target Genes Regulated by AP-2 Transcription Factors

Target lists for AP-2γ and AP-2α were compiled using the following databases: Gene Transcription Regulation Database (GTRD; v19.10), TRANScription FACtor database (TRANSFAC; v2019.2) and Transcriptional Regulatory Relationships Unraveled by Sentence-based Text mining (TRRUST; v2). Excluding duplicates, 5175 targets were identified for AP-2γ and 4810 for AP-2α.

### 2.6. Differential Expression Analysis

The R-package limma (v3.42.2) was used to perform differentiation expression analysis, with voom function to make CAGE-seq data useful for limma. Data were preprocessed using calcNormFactors() and low expressed genes were filtered out (tags that had at least 5 counts per million in at least 1 library were retained). Following voom transformation, the model was fitted in limma using weighted least squares for each gene via lmFit(). Log_2_ fold-change (log_2_FC) for each comparison between groups was obtained with the use of makeContrasts(), default parameters. Empirical Bayesian smoothing of standard errors was used prior to extraction of the top-ranked genes using topTable().

### 2.7. Identification of Genes Inversely Regulated by WWOX and AP-2 and Their Functional Annotation

**Figure 2:**
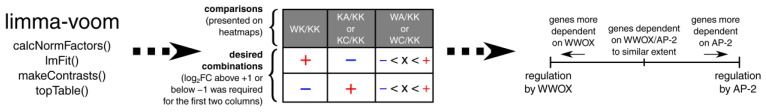
Graphical explanation of the methodology step performed to identify inversely regulated genes across cellular variants.

Genes from differential expression analysis were temporarily limited to AP-2α or AP-2γ targets, depending on comparison. KA, WK and WA were compared to KK (henceforth called ‘alpha’ comparisons) via AP-2α target list while KC, WK and WC were compared to KK (henceforth called ‘gamma’ comparisons) through AP-2γ equivalent. These filtered lists were then input into R environment and rows having opposite marks in two values (of two different columns/phenotypes) were retained using sign() function, which was followed by verification of whether a value in another (third) column is between two previous ones. Requirement of log_2_FC above 1 or below −1 had to be met for the values in the first and second columns. Through that, it was possible to keep AP-2 targets whose expression is inversely regulated by WWOX and AP-2α/γ, which is alleviated when these two are overexpressed within a single cellular variant. A schematic presentation of this step is summarized in Figure 2. Heatmaps were generated using the Shiny web server Heatmapper (http://www.heatmapper.ca/; accessed on 9 November 2021). The row scale type was set along with the average linkage clustering method and Pearson distance measurement. Clustering to rows was applied to visualize the dendrogram.

The observed relationships were verified on the protein level using Western blot (two representatives were selected), see section “Protein extraction and Western blot” for details. Subsequently, the targets were functionally annotated in parallel using Pathview Web (auto-selection of implicated pathways from Kyoto Encyclopedia of Genes and Genomes (KEGG)) and The Database for Annotation, Visualization and Integrated Discovery (DAVID; “KEGG_PATHWAY” module) in order to preliminarily identify the signaling in which these genes might be involved, and whether certain pathways repeat between ‘alpha’ and ‘gamma’ comparisons. Additionally, the Epithelial–Mesenchymal Transition Gene Database (dbEMT) was used along with Pathview Web and DAVID (accessed on 17 December 2021) to identify the KEGG pathways containing the greatest number of EMT-related genes (“browse” and “pathway” options were executed and the top KEGG charts were then selected).

### 2.8. Pathway Visualization and Integration

Recognized pathways from previous step were visualized using the pathview() function (v1.26.0) in R environment as KEGG native graphs with limit value of 3 for gene data when converting to pseudocolors and bins parameter = 20. After careful analysis of the obtained results, it was decided to use Inkscape, integrating all relevant data from pathways or literature data to the form of single graph that can explain the main molecular differences/similarities between phenotypes and link them to biological processes. The observed relationships were verified on the protein level using Western blot (five representatives were selected), see Section 2.2 for details.

### 2.9. Protein Association Networks Supported by Molecular Signatures

The Molecular Signatures Database (MSigDB) and Gene Reference into Function (GeneRIF) resources (accessed on 26 January 2022) were browsed to select gene sets implicated in aforementioned biological processes. Altogether, 13 datasets were chosen for collection, i.e., CAGE-seq data were filtered to retain only genes implicated in selected MSigDB/GeneRIF terms (Table 1). Afterwards, the top 50 genes based on log_2_FC (the top 25 most upregulated and the top 25 most downregulated) from each comparison and each biological process were treated as an input to network visualization through Cytoscape software (v3.8.0) using the Search Tool for the Retrieval of Interacting Genes/Proteins (STRING) protein query option (v1.5.0), default parameters. Similarly, the definitive network was prepared from the top 3 most upregulated and the top 3 most downregulated genes per comparison in all biological processes using GeneMania plugin (v3.5.2) of Cytoscape (automatic network weighting and zero “resultant” genes). The definitive network was supported by intersection analysis; the Venn diagram was prepared with the use of the venn() function (v1.10) and further modified visually using Inkscape (v1.1) to include item labels.

### 2.10. Principal Component Analysis

Genes of definitive networks were used as a set for dimensional grouping performed via principal component analysis (PCA). All 173 genes were involved as active variables and their contribution in spatial partitioning across PC1 and PC2 components was visualized via FactoMineR plugin (v1.7) of Rcmdr(), as well as factoextra().

## 3. Results

### 3.1. WWOX Can Overexpress or Downregulate the Genes Which Are Inversely Regulated by AP-2 Factor

**Figure 3:**
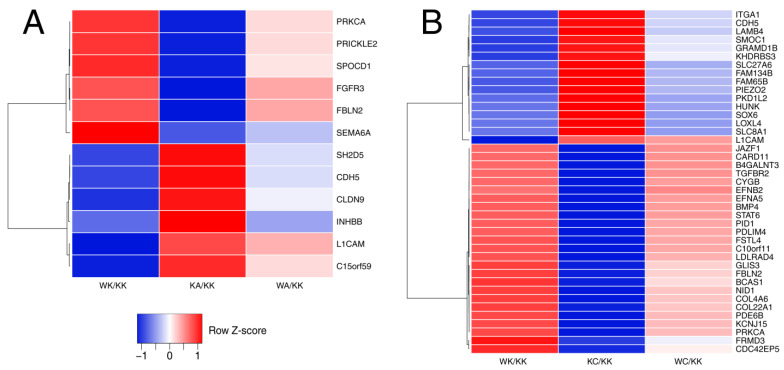
Heatmaps presenting inversely regulated AP-2 targets across (**A**) ‘alpha’ and (**B**) ‘gamma’ comparisons.

The targets of AP-2 transcription factors were first filtered to retain the genes whose expression was regulated in opposite directions by WWOX (WK) and AP-2α (KA) or AP-2γ (KC) when compared to control (KK). Subsequently, it was verified whether the difference in expression is alleviated in the rescue variant (i.e., WA or WC, depending on comparison). Thus, the expression of each AP-2 target in the WA variant was between that of WK and KA, as was the case of WC in reference to WK and KC. As visible in Figure 3, the above requirements were met by less genes in ‘alpha’ comparisons (Figure 3A) than in ‘gamma’ counterparts (Figure 3B). ‘Alpha’ or ‘gamma’ stems from AP-2α or AP-2γ, respectively, and refers to all variants that are compared in order to disclose the role of a specific AP-2 factor as well as WWOX (‘alpha’ comparisons are visible in Figure 3A while ‘gamma’ comparisons in Figure 3B). In both cases, it is possible to identify genes which are more dependent on WWOX (e.g., *INHBB* for ‘alpha’ and *EFNB2* for ‘gamma’ comparisons) or AP-2 factor (*SEMA6A* for AP-2α and *L1CAM* for AP-2γ). Nevertheless, there are also examples where a gene is similarly dependent on WWOX and AP-2, e.g., *CLDN9* in ‘alpha’ and *KHDRBS3* in ‘gamma’ comparisons. Some targets were identified in two sets, i.e., *CDH5*, *FBLN2*, *L1CAM* and *PRKCA*, and their changes in expression indicate that they are similarly regulated by both AP-2α and AP-2γ.

### 3.2. Many Important Pathways Were Denoted, with Rap1 and MAPK Repeating between Comparisons

All genes from heatmaps were functionally annotated to KEGG pathways. Based on data combined from Pathview Web and DAVID, apart from annotations that referred to processes, there were some associated with signal transduction. Respectively for ‘alpha’ or ‘gamma’ comparisons, the identified signaling pathways were Wnt, Rap1, MAPK or PI3K-Akt, Rap1, MAPK, Ras. Due to the fact that signal transduced by these pathways is implicated in epithelial-to-mesenchymal transition (EMT) and that this process is not covered in KEGG as a dedicated map, the dbEMT webtool was additionally used to identify KEGG pathways that contain the greatest number of EMT-related genes. Equally, hsa05206 (MicroRNAs in cancer) and hsa05200 (pathways in cancer) contained 170 genes related to EMT while hsa04151 (PI3K-Akt signaling pathway) was next in order, with 97 genes implicated in this process. Since the PI3K-Akt was already identified through Pathview/DAVID, and “Pathways in cancer” was one of processes annotated for ‘gamma’ comparisons, it was decided to include “MicroRNAs in cancer” for both ‘alpha’ and ‘gamma’ comparisons and “Pathways in cancer” for the ‘alpha’ comparison. The KEGG pathways subjected to further analysis are summarized in Table 2.

**Figure 4:**
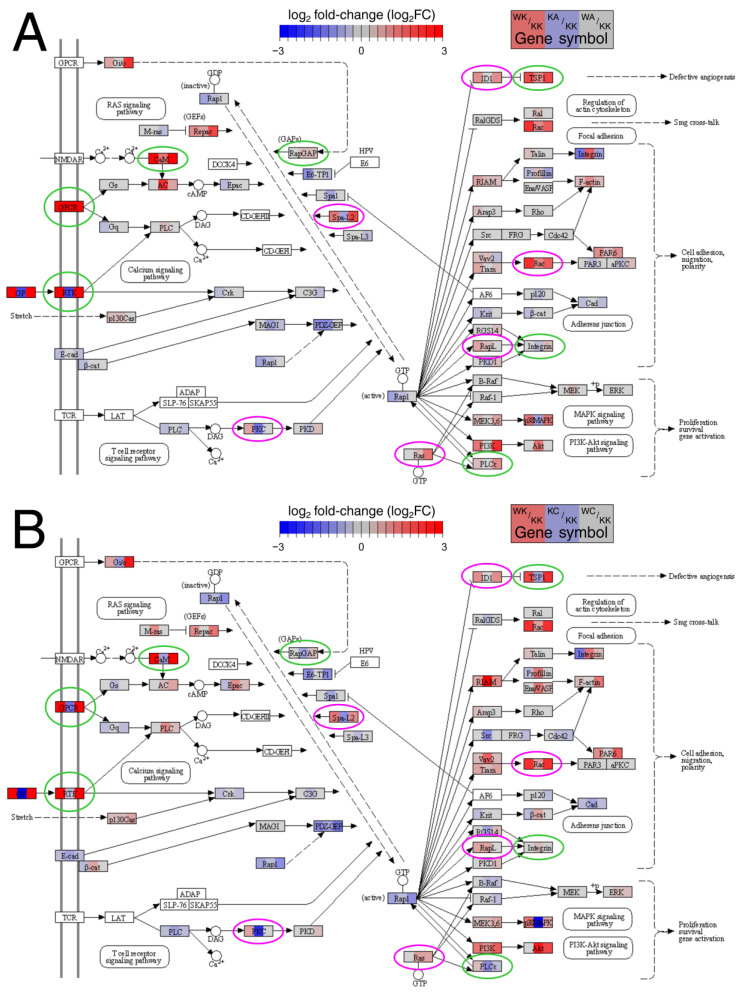
Example differences and similarities in the Rap1 signaling pathway between (**A**) ‘alpha’ and (**B**) ‘gamma’ comparisons. Differences are marked in green while similarities in pink.

Afterwards, the above pathways were visualized separately for ‘alpha’ and ‘gamma’ comparisons. In this step, the temporary restriction of investigating only AP-2 targets was lifted; thus, all genes from differential expression analysis were included. An example pathway which repeated in ‘alpha’ (Figure 4A) and ‘gamma’ (Figure 4B) comparisons was Rap1 signaling. Some changes and similarities in gene expression were observed. Namely, while the expression of G Protein-Coupled Receptors (GPCRs), calmodulins (CaMs) and thrombospondin 1 (TSP1) was increased during WWOX or AP-2α overexpression, the opposite was found when AP-2γ was overexpressed. In contrast, the regulation of Spa-L2 was similar during AP-2α and AP-2γ overexpression compared to WWOX. Likewise, the Rac proteins had the same pattern of expression changes in ‘alpha’ and ‘gamma’ comparisons. Lastly, although the changes in Receptor Tyrosine Kinases (RTKs) were in the same direction for AP-2γ and AP-2α, the latter decreased their expression to a greater extent. However, regardless of AP-2 factor, the regulation of aforementioned examples in rescue variants seems to be WWOX-dependent (WA/KK and WC/KK patterns are similar to WK/KK). The remaining pathways for ‘alpha’ and ‘gamma’ comparisons are visualized in Figures S1 and S2, respectively.

### 3.3. Altered Signaling Was Found to Be Involved in the Regulation of Multiple Biological Processes

**Figure 5:**
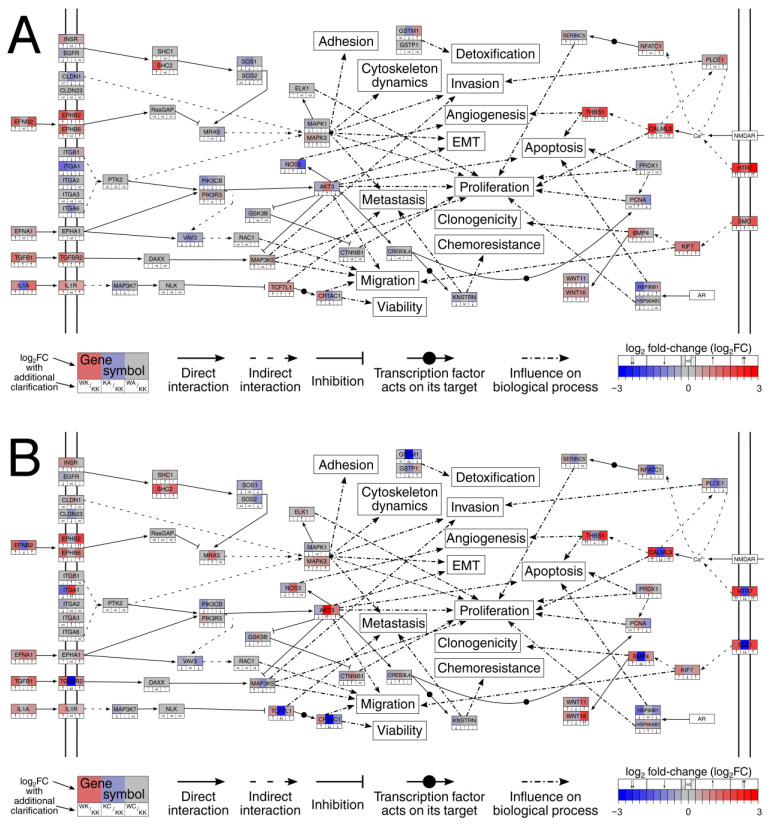
Differences and similarities between (**A**) ‘alpha’ and (**B**) ‘gamma’ comparisons regarding the signal transduction.

Due to the extensiveness of information obtained from KEGG graphs, it was intended to integrate all relevant data to the form of a single graph in order to collate the molecular differences and similarities between phenotypes, linking them to biological processes (Figure 5). About half of the processes were previously investigated in our in vitro research, the remainder was included to capture the essence of the carcinogenesis process from the literature data on genes that were included in the graph. For instance, the signal transduction beginning with *TGFB1* and *TGFBR2* could affect invasion, proliferation or migration processes through *MAP3K5*. In ‘alpha’ comparisons (Figure 5A) the changes caused by either WWOX or AP-2α overexpression are consistent with each other, i.e., WWOX increased *TGFB1*, *TGFBR2* and *MAP3K5* expression while AP-2 increased the first two but did not affect the latter. On the contrary, changes visible in ‘gamma’ comparisons (Figure 5B) indicate that AP-2γ decreased *TGFB1*, *MAP3K5* and *TGFBR2* (herein listed in the order of potentiated downregulation). Likewise, the signaling by *SMO*, *KIF7* and *BMP4* is decreased by AP-2γ but increased by WWOX and AP-2α, which could affect migration or clonogenicity.

### 3.4. Observations Were Validated on the Protein Level

**Figure 6:**
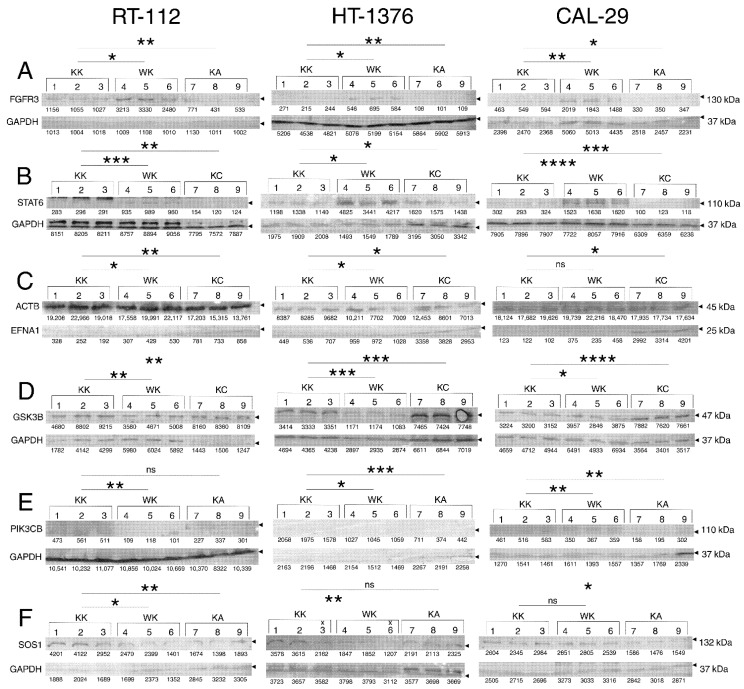
Validation of selected CAGE-seq observations on the protein level. (**A**) FGFR3. (**B**) STAT6. (**C**) EFNA1. (**D**) GSK3B. (**E**) PIK3CB. (**F**) SOS1. Stars represent statistical significance; *p*-value < 0.05 (*), *p*-value < 0.01 (**), *p*-value < 0.001 (***), *p*-value < 0.0001 (****).

In order to certify the observations from the previous sections, some representatives were selected for validation at the protein level using Western blot (Figure 6). Two representatives (FGFR3, STAT6) were selected from Figure 3 (one per heatmap) and five representatives (EFNA1, GSK3B, PIK3CB, SOS1, KIF7) from Figure 5. Due to the fact that the difference in expression is frequently alleviated in rescue variants, it was decided to focus on changes caused by WWOX overexpression (WK) or AP-2α/γ overexpression (KA or KC) when compared to control (KK). For each protein, the differences in comparisons were statistically significant in at least two cell lines. The blotting results for FGFR3 were found to be in line with CAGE-seq data, i.e., expression is increased in WK variant but decreased in KA when compared to KK, as determined in all investigated cell lines (respectively for RT-112, HT-1376 and CAL-29, the mean expression for KK was 1.0669 ± 0.0678, 0.0500 ± 0.0025 and 0.2223 ± 0.0291; for WK was 2.8808 ± 0.3800, 0.1182 ± 0.0137 and 0.3675 ± 0.0318; for KA was 0.5468 ± 0.1287, 0.0179 ± 0.0007 and 0.1432 ± 0.0123). Similar concerns the STAT6: while the protein level is higher in WK than in KK, it is lower in KA than in KK; the most evident observations were noted in the CAL-29 cell line (respectively for RT-112, HT-1376 and CAL-29, the mean expression for KK was 0.0355 ± 0.0006, 0.6252 ± 0.0684 and 0.0388 ± 0.0019; for WK was 0.1081 ± 0.0028, 2.6025 ± 0.5481 and 0.2017 ± 0.0039; for KC was 0.0172 ± 0.0023, 0.4847 ± 0.0472 and 0.0180 ± 0.0019). The greatest difference in expression was again visible in CAL-29 regarding EFNA1 for which increased protein level was noted in KC compared to KK; the WK/KK comparison did not meet statistical significance in this cell line, yet it was met in the other two (respectively for RT-112, HT-1376 and CAL-29, the mean expression for KK was 0.0127 ± 0.0038, 0.0638 ± 0.0094 and 0.0063 ± 0.0009; for WK was 0.0209 ± 0.0032, 0.1223 ± 0.0267 and 0.0181 ± 0.0071; for KC was 0.0519 ± 0.0092, 0.3786 ± 0.0951 and 0.1973 ± 0.0368). However, WK and KC affected GSK3B expression in opposite directions which is prominent in all cell lines (respectively for RT-112, HT-1376 and CAL-29, the mean expression for KK was 2.2982 ± 0.2843, 0.7606 ± 0.0318 and 0.6696 ± 0.0284; for WK was 0.7414 ± 0.1291, 0.3938 ± 0.0148 and 0.5818 ± 0.0257; for KC was 5.9008 ± 0.5238, 1.1059 ± 0.0223 and 2.2098 ± 0.0309). In the case of PIK3CB expression, it was challenging to spot unequivocal changes yet the protein level decreased for both WK and KA; the decrease in KA compared to KK did not meet statistical significance in RT-112 (respectively for RT-112, HT-1376 and CAL-29, the mean expression for KK was 0.0486 ± 0.0054, 0.9752 ± 0.0900 and 0.3657 ± 0.0324; for WK was 0.0105 ± 0.0012, 0.6297 ± 0.3331 and 0.2373 ± 0.0236; for KA was 0.0305 ± 0.0094, 0.2270 ± 0.0762 and 0.1189 ± 0.0096). A similar finding, yet still consistent with CAGE-seq data, was noted for SOS1 for which observations from the RT-112 cell line were the most valuable (respectively for RT-112, HT-1376 and CAL-29, the mean expression for KK was 2.0025 ± 0.2403, 0.8510 ± 0.2147 and 1.0034 ± 0.1254; for WK was 1.1669 ± 0.2483, 0.4542 ± 0.0575 and 0.8538 ± 0.0808; for KA was 0.5314 ± 0.0858, 0.6060 ± 0.0317 and 0.5288 ± 0.0356). On the other hand, it was impossible to detect bands that correspond to KIF7.

### 3.5. Definitive Network Was of High Interconnectivity and Phenotypes Were Explained by Dimensional Grouping

The genes from differential expression analysis were filtered to retain only those that are involved in the regulation of the processes summarized in Figure 5. Based on the fold-change, the top 50 genes from each comparison (WK/KK, KA/KK, KC/KK, WA/KK, WC/KK) that are related to each biological process were visualized in the form of a protein–protein interaction network. This resulted in five networks (one per comparison), each containing top genes for 13 processes (Figure S3). At first glance, there are genes related to only one specific process but also representatives of a mixed group whose role is not limited to a single phenomenon; they often allowed protein–protein interactions to be linked between processes. A small group of proteins that did not interact with others (so-called “singletons”) is also present; however, each network still demonstrated high interconnectivity.

**Figure 7:**
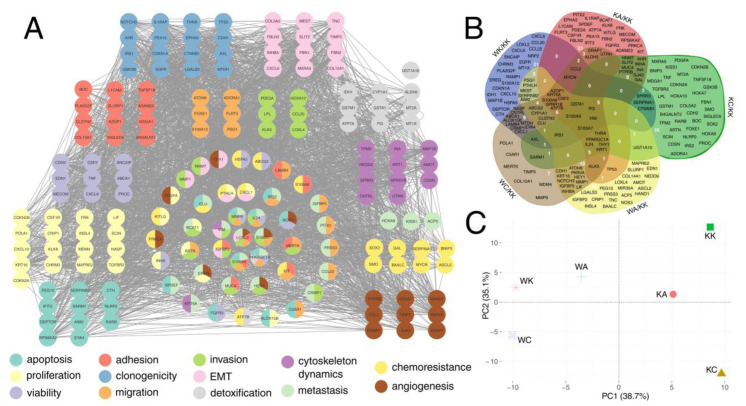
Visualization of definitive network and the PCA of cellular variants using gene expression data. (**A**) Definitive network with emphasis on processes. (**B**) Intersection analysis of definitive network. (**C**) PCA using expression data of genes from definitive network.

Ultimately, the above networks were collated to form a single network containing data for all comparisons and processes. For this purpose, only the top 3 most upregulated and the top 3 most downregulated genes for each biological process within each comparison were included. The content of the definitive graph with emphasis on processes (Figure 7A) or comparisons (Figure 7B) indicated that except *UGT1A10*, all genes comprised one network, proving that this set of the most up- or downregulated genes could influence all aforementioned processes as a whole. The entire set was input to PCA analysis to verify whether various expression of these genes among cellular variants could explain phenotypes. On the one hand, the differences were observed between variants with and without WWOX overexpression; on the other, variants with AP-2γ overexpression were distinct from the others (Figure 7C).

## 4. Discussion

HTS has revolutionized cancer research [16] but for conclusive evidence it is preferred to use the biological replicates in order to limit the impact of tumor heterogeneity [17,18]. The diverse signaling is an example of what underlies the heterogeneity [2,3]; thus, the aim of this research was to use three BLCA cell lines as biological replicates in an attempt to investigate the influence of WWOX, AP-2α and AP-2γ on the signal transduction in this tumor. Investigating the molecular pathways could clarify the otherness of phenotypes as well as contribute to cancer treatment [3].

To determine the role of WWOX in cellular pathways activated by AP-2α/γ, the inversely regulated AP-2 targets were first identified. The results indicated that some genes could be similarly dependent on WWOX and AP-2 factor while others could be regulated to a greater degree by one or the other. These findings, particularly the inverse regulation by WWOX and AP-2 TFs, have not been noted in previous studies; however, *PRKCA* repression by AP-2γ [19] or *FBLN2* downregulation by AP-2α [20] have been noted. Moreover, *BMP4* was found to be positively correlated with WWOX [21], which is in line with *BMP4* upregulation in the WWOX-overexpressing variant in the present study.

The identification of AP-2 targets and their functional annotation provided an insight into the signaling pathways in which they are involved. The data on the role of WWOX, AP-2α and AP-2γ in the regulation of MAPK [22,23,24], Wnt [25,26,27], Ras [28,29,30] and PI3K-Akt [31,32,33] signaling are available; however, no influence on Rap1 signaling was found in the literature, suggesting that these could be novel observations. Aside from signal transduction, WWOX and two AP-2 factors are implicated in adhesion [34,35,36], migration [24,37,38], axon guidance (indirectly) [39,40,41] and regulation of extracellular matrix [42,43,44] or miRNA [45,46]. Afterwards, these pathways/processes were visualized as KEGG graphs to present differences and similarities between ‘alpha’ and ‘gamma’ comparisons. Apart from evident examples such as *THBS1* (TSP1) in Rac1 signaling, which is responsible for angiogenesis inhibition [47] and was found to be upregulated by WWOX and AP-2α but downregulated by AP-2γ, the analysis of the other ones was not that convenient. This is due to the number of graphs or to the fact that KEGG charts collect expression data from gene groups into one object (the single rectangle is not always a single gene). Therefore, we set ourselves the goal of drawing a collective signal transduction graph that would explain the differences in expression of specific genes and show the further consequences regarding biological processes. In the two subsequent paragraphs, these genes are discussed in sequence.

Beginning with signal transduction via *HTR7*, the downstream changes in *CALML3* and *THBS1* affect angiogenesis and apoptosis while the alterations in *PLCE1*, *NFATC1* and *SERINC5* influence invasion or proliferation. *CALML3* was found to be downregulated during tumor progression [48] which would explain its increased level during WWOX overexpression but decreased level during AP-2γ overexpression. Data from other reports also indicate that high *CALML3* decreases proliferation [49,50]. As mentioned previously, *THBS1* is an anti-angiogenic molecule [47] but its function is also manifested through pro-apoptotic signals [51]. *THBS1* increase may inhibit angiogenesis and stimulate apoptosis during WWOX or AP-2α overexpression, contrary to AP-2γ which downregulates this gene. A similar finding applies to *PLCE1* which prevents the tumor invasion [52]; it is increased during WWOX overexpression but not when AP-2γ is at a high level. Through Ca^2+^-dependent signaling, *PLCE1* affects *NFATC1* which further regulates *SERINC5*. The latter is best known for its ability to inhibit viral infections [53]; however, Bossolasco et al. indicated that *SERINC5* is responsible for transition of cells from a proliferative to a postmitotic state [54]. During such events, the downregulation of genes involved in the mitogenic response is observed [55]. This suggests that *SERINC5* can inhibit proliferation when highly expressed, which would occur during WWOX overexpression. Intriguingly, it is downregulated by both AP-2 factors, though more by AP-2γ. The next signaling begins with *SMO* and regulates migration via *KIF7* or clonogenicity via *BMP4*. The high *KIF7* inhibits migration [56] which is probably a case in variants with WWOX or AP-2α overexpression, unlike in AP-2γ where expression of this gene is decreased. Likewise, the high *BMP4* limits tumor clonogenicity [57] and the influence of WWOX and AP-2 factors on *BMP4* expression is similar to that of *KIF7*. *BMP4* also transduces a signal to *WNT11* or *WNT16*, both of which were found to be elevated during WWOX or AP-2α overexpression or reduced in the variant overexpressing AP-2γ. The reduction in *WNT11* is associated with bladder tissue fibrosis [58], and this phenomenon may elicit the initiation of BLCA [59]. Regarding *WNT16*, the improved patient’s prognosis was observed when this gene was highly expressed [60]. Another signaling which influenced apoptosis and proliferation was through *HSP90B1* and *HSP90AB1*. The former was decreased during WWOX or AP-2α overexpression but was not regulated by AP-2γ; it appears that downregulation of *HSP90B1* by WWOX/AP-2α would induce apoptosis of cancer cells [61]. The other chaperone, *HSP90AB1*, was mainly decreased by WWOX but increased by AP-2γ. Literature data indicate that it is overexpressed during carcinogenesis and presumably potentiates the proliferation [62]. Two glutathione S-transferases were also noticed, namely the *GSTM1* and *GSTP1*. Although neither of the two were evidently regulated only by WWOX, both AP-2 factors were involved in the changes in their expression. The downregulation of *GSTM1* is thought to be a biomarker of bladder carcinogenesis [63] and our data indicate that while both AP-2 factors decrease its expression, AP-2γ does so to a greater extent. However, while the downregulation of *GTSP1* is also not beneficial (it leads to cancer progression [64]), its expression is only reduced by AP-2γ and not AP-2α.

The changes in signaling that begin with *IL1A* and *IL1R* interaction had an impact on metastasis and viability or migration via *TCF7L1* or *CRTAC1*. Based on the literature, the role of *TCF7L1* is equivocal but some research indicate that it acts as a tumor suppressor [65] and inhibits metastasis [66]. Thus, it was decided to search for an appropriate target of this transcription factor to certify its anti-cancer characteristics specifically in BLCA. It turned out that *CRTAC1* is of interest for bladder cancer since it inhibits many pro-tumorigenic processes including cell viability and migration [67]. To recapitulate, the decrease in both *IL1A* and *MAP3K7* (during AP-2α overexpression) or only *MAP3K7* (during WWOX overexpression) can further lead to an increase in *TCF7L1*, which affects metastasis and *CRTAC1* expression. However, *CRTAC1* might also be regulated by other mechanisms since both AP-2α and AP-2γ downregulate its expression (the latter to a greater extent). On the other hand, no decrease in *IL1A* or *MAP3K7* is observed during AP-2γ overexpression, but *TCF7L1* and *CRTAC1* are greatly downregulated, promoting the aforementioned processes. The other signal was transduced via *TGFB1* and *TGFBR2* to *MAP3K5*; all three genes were upregulated by WWOX overexpression, similar to AP-2α which increased the first two. However, high AP-2γ decreased *TGFBR2* and *MAP3K5*; the reduction in these two genes potentiates proliferation, migration and invasion [68,69,70]. Moving forward, *RAC1* was the downstream effector of *VAV3* which was activated by interaction of *EFNA1* and *EPHA1*. Although no evident changes between variants were noticed in *RAC1* or *EPHA1*, the *VAV3* gene was downregulated during WWOX or AP-2α, but not during AP-2γ overexpression; this suggests that no reduction in migration occurs with the latter. However, *EFNA1* is increased when AP-2γ is highly expressed, which could be associated with resistance to cancer treatment [71]. This is intriguing since WWOX also moderately increased *EFNA1* expression but because *VAV3* is both decreased and downstream of the signaling, it is possible that it does not manifest as in AP-2γ overexpression. Another signaling which started with interaction of *EFNB2* and *EPHB2* or *EPHB6* ended with *MAPK1*, *MAPK3* or *ELK1*. Here, the expression of *EFNB2*, *EPHB2* or *EPHB6* was increased by WWOX, similar to AP-2α (which elevated *EPHB2*) but contrary to *AP*-2γ (which mainly decreased *EFNB2* and *EPHB2*). *EFNB2* is responsible for inhibition of the invasion [72] while receptors *EPHB2* and *EPHB6* are thought to be downregulated during successive steps of carcinogenesis [73,74]. No prominent differences in *MAPK1* and *MAPK3* expression were observed between ‘alpha’ and ‘gamma’ comparisons, suggesting that upstream changes are more of interest; for example, *MRAS* can increase proliferation [75] and was elevated by AP-2γ but was not regulated by WWOX or AP-2α. This complements the data on *ELK1* which is thought to potentiate mitogenic gene expression [76] and was also increased only by AP-2γ. Two claudins, *CLDN1* and *CLDN23*, were also indirectly connected with the above signaling. The WWOX overexpression did not affect their level but contrasting results were observed between AP-2α and AP-2γ regarding *CLDN1* expression. Literature data indicate that high *CLDN1* increases invasion and EMT [77,78]; this gene is upregulated by AP-2γ but downregulated by AP-2α. Moreover, the latter reduced the expression of *CLDN23*, whose downregulation is thought to be a frequent event in carcinogenesis (at least in gastric cancer) [79]. *MRAS* was also downstream of the signal transduced by *INSR* and *EGFR* and subsequently *SHC1*/2 and *SOS1*/2. The finding of increased *SHC1*/2 during AP-2γ overexpression could be a reason for elevated *MRAS*. On the other hand, the lack of *MRAS* upregulation during WWOX or AP-2α overexpression could result from the downregulation of *SOS1* or *SOS2*. The last signaling was transduced by integrins; collectively, they were either decreased or not regulated by WWOX or AP-2α, contrary to AP-2γ which either increased or did not regulate the genes from this group. Increased *ITGB1* results in potentiated proliferation, migration, invasion or decreased apoptosis [80], similar to elevated *ITGA1* which leads to proliferation or invasion [81]. The remaining integrins of interest, during their overexpression, are responsible for inhibition of both apoptosis and response to therapy (*ITGA2*) [82], increase in proliferation (*ITGA3*) [83] or invasion and metastasis intensification (*ITGA6*) [84]. Furthermore, *PIK3CB* and *PIK3R3* are both downregulated during AP-2α overexpression but not during high AP-2γ; these two genes induce metastasis and EMT, respectively [85,86]. The increase in *PIK3R3* during WWOX overexpression is questionable but still the genes that are downstream in signaling are downregulated, i.e., *VAV3* (discussed above) and *AKT3*. Regarding the latter gene, it was found to be downregulated by WWOX but upregulated by AP-2α and even more by AP-2γ; high *AKT3* triggers proliferation but reduces apoptosis [87,88]. *AKT3* directly acts on *NOS3* which regulates angiogenesis [71] which is probably reduced by WWOX, marginally increased by AP-2α, and notably elevated by AP-2γ. A similar observation applies to *CREB3L4*, the transcription factor regulating *KNSTRN* which regulates metastasis and chemoresistance [89] or *PCNA* which regulates proliferation [90]. WWOX downregulated *CREB3L4* and *KNSTRN* while AP-2α only marginally increased *PCNA*. With AP-2γ overexpression, *CREB3L4* and *PCNA* were upregulated. The *PCNA* gene is also influenced by *PRDX1*, which promotes proliferation but inhibits apoptosis when overexpressed [91]; *PRDX1* is elevated by AP-2γ but decreased by WWOX. The last two genes, *GSK3B* and *CTNNB1*, were downregulated by WWOX but upregulated by AP-2γ (no difference in expression was seen during AP-2α overexpression). Along with *AKT3*, they repress one another but seem to also be regulated by other mechanisms since high *AKT3* level during AP-2γ did not inhibit *GSK3B*, similar to *CTNNB1*. Nevertheless, the last two increase proliferation when overexpressed [92,93] which certifies the above data on WWOX or AP-2γ.

**Figure 8:**
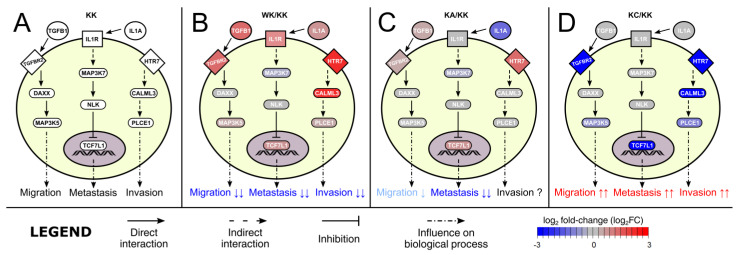
The most relevant similarities and differences in signaling by WWOX, AP-2α and AP-2γ. (**A**) KK variant. (**B**) WK/KK comparison. (**C**) KA/KK comparison. (**D**) KC/KK comparison.

Overall, the signal transduction findings indicate that WWOX and AP-2α regulate processes in a similar manner, but different from AP-2γ. Phenotypically, this has been noticed previously [6,7]. Based on the data from the above paragraphs, we aimed to choose the most relevant signaling that could evidently illustrate similarities and differences, taking into account the impact on related processes. It turned out that there were evident changes in migration, as well as metastasis and invasion (Figure 8). Compared to control variant, WWOX overexpression inhibited the aforementioned processes while completely opposite observations were noticed for AP-2γ. In terms of AP-2α, the metastasis inhibition was mainly due to *TCF7L1* (similar to WWOX); migration could also be moderately decreased via *TGFBR2* overexpression but no changes were identified for *MAP3K5*. Moreover, the influence of AP-2α on invasion is not evident as the expression of a direct effector, *PLCE1*, is not modulated (and the increase in only *HTR7* might not be sufficient). Nevertheless, the resemblance of WWOX and AP-2α is visible. From another perspective, one could also analyze these three processes as the essence of EMT [94,95]. Literature data indicate that WWOX is responsible for EMT inhibition [96] while AP-2γ may enhance this process [24]. As for AP-2α, it most probably stimulates the mesenchymal-to-epithelial transition (MET) [97]. Our phenotypic observations have suggested this previously [7], but these findings complement them by explaining the molecular background.

*FGFR3*, *STAT6*, *EFNA1*, *GSK3B*, *PIK3CB*, *SOS1* and *KIF7* were chosen as representatives for validation of CAGE-seq at the protein level using Western blot. Except for *KIF7*, for which no bands were detected, the remaining representatives certified the sequencing data in the investigated cell lines. While most of these proteins are components of pathways that are regulated by WWOX or AP-2α/γ (e.g., FGF [98,99], PI3K [31,32,33] and JAK/STAT [21,100] signaling), only regulation of *GSK3B* could be individually confirmed by literature data. Similar to observations from the current study, WWOX was found to repress GSK3B activity; such outcome results from direct binding of WWOX to GSK3B [101,102].

The last part of the analysis concerned the identification of the genes which are implicated in the aforementioned biological processes. Initially, one network per comparison (WK/KK, KA/KK, KC/KK, WA/KK, WC/KK) was constructed. Following this, a definitive network was created using the top 3 most upregulated and the top 3 most downregulated genes for each biological process within each comparison. This resulted in a group of 173 genes that was of high interconnectivity, except for *UGT1A10* which was a singleton. Thus, the network which consisted of the most up- or downregulated genes could influence all the aforementioned biological processes. To complement these results with observations from previous research, example genes were used to justify the phenotypic differences between variants. For instance, high *CXCL10* is implicated in proliferation impairment [103] and the expression of this gene in the current study was increased in WK, which was the variant with decreased proliferation [7]. The next example is *MECOM* which is responsible for maintaining cell viability [104]; the downregulation of this gene by high AP-2α could therefore limit viability, as previously evidenced in the KA variant [6,7]. *GSK3B*, being increased by AP-2γ, can escalate clonogenicity [105] and the previous studies have noted that the KC variant has more colonies [6,7]. The downregulation of *PEG10* in the WA variant is probably to restore apoptosis since this gene is implicated in cell death inhibition [106]; one could see increased apoptosis in the WA variant [6]. The last example, *MMP9*, enhances invasion [107] and was found to be upregulated in the WC variant, as in a previous study determined by gelatin zymography [7]. PCA using the genes from the definitive network indicated that cellular variants differ in the expression of these genes. This is in line with conclusions from previous research [7] where the net effect observed in WA is a combination of that of WK and KA. Likewise, KC and WC were distinct phenotypes when compared to their non-AP-2γ-overexpressing equivalents.

## 5. Conclusions

To conclude, the current study complements the up-to-date findings on WWOX and AP-2α/γ in BLCA and indicates genes that are worth investigating with regard to the broad spectrum of included biological processes. Evident differences in signal transduction were noted for migration, metastasis and invasion via TGFBR2/MAP3K5, MAP3K7/TCF7L1 and HTR7/PLCE1 signaling, respectively. Thus, previous phenotypic observations that supposedly WWOX is an EMT inhibitor, AP-2γ an EMT enhancer while AP-2α an MET inducer, were found to be explainable at the molecular level in bladder cancer. The study also shows the gene expression changes depending on the level of WWOX and AP-2α or AP-2γ; this confirms previous phenotypic observations and indicates that WWOX and AP-2α have more in common than AP-2α and AP-2γ. Some of the identified genes can find application in bladder cancer treatment; likewise, AP-2γ appears to offer promise in targeted therapy aimed at transcription factors.

## Figures and Tables

**Table 1:** Gene sets selected to represent biological processes.

Biological Process	Gene Set Accession Number or Direct Link
**MSigDB**	
Apoptosis	GO:0006915
Proliferation	GO:0008283
Adhesion	GO:0022610
Migration	GO:0016477
Epithelial-to-Mesenchymal Transition	M5930
Cytoskeleton dynamics	GO:0005856
**GeneRIF**	
Viability	https://maayanlab.cloud/Harmonizome/gene_set/ viability/GeneRIF + Biological + Term + Annotations (accessed on 26 January 2022).
Clonogenicity	https://maayanlab.cloud/Harmonizome/gene_set/ clonogenicity/GeneRIF + Biological + Term + Annotations (accessed on 26 January 2022).
Invasion	https://maayanlab.cloud/Harmonizome/gene_set/ invasion/GeneRIF + Biological + Term + Annotations (accessed on 26 January 2022).
Detoxification	https://maayanlab.cloud/Harmonizome/gene_set/ detoxification/GeneRIF + Biological + Term + Annotations (accessed on 26 January 2022).
Metastasis	https://maayanlab.cloud/Harmonizome/gene_set/ metastasis/GeneRIF + Biological + Term + Annotations (accessed on 26 January 2022).
Chemoresistance	https://maayanlab.cloud/Harmonizome/gene_set/ chemoresistance/GeneRIF + Biological + Term + Annotations (accessed on 26 January 2022).
Angiogenesis	https://maayanlab.cloud/Harmonizome/gene_set/ angiogenesis/GO + Biological + Process + Annotations (accessed on 26 January 2022).

**Table 2:** KEGG pathways from functional annotation of AP-2 targets in ‘alpha’ and ‘gamma’ comparisons. Underlined and in bold are pathways repeated in Pathview Web and DAVID. Comp.—comparison.

Comp.	Pathview Web	DAVID	dbEMT
Alpha	MAPK signaling pathway (hsa04010); **Cell adhesion molecules (hsa04514);** **Leukocyte transendothelial migration (hsa04670)**	Wnt signaling pathway (hsa04310); Rap1 signaling pathway (hsa04015); Signaling pathways regulating pluripotency of stem cells (hsa04550) **Cell adhesion molecules (hsa04514);** **Leukocyte transendothelial migration (hsa04670);** Axon guidance (hsa04360);	Pathways in cancer (hsa05200); MicroRNAs in cancer (hsa05206)
Gamma	**PI3K-Akt signaling pathway (hsa04151);** MAPK signaling pathway (hsa04010); **Focal adhesion (hsa04510)**	**PI3K-Akt signaling pathway (hsa04151);** Rap1 signaling pathway (hsa04015); Ras signaling pathway (hsa04014); **Focal adhesion (hsa04510);** ECM-receptor interaction (hsa04512); Protein digestion and absorption (hsa04974); Amoebiasis (hsa05146); Axon guidance (hsa04360); Pathways in cancer (hsa05200)	MicroRNAs in cancer (hsa05206)

## Data Availability

The datasets generated and/or analyzed during the current study are available in the NCBI GEO repository, GSE193659.

## Supplementary Materials

The following are available online at https://www.mdpi.com/article/10.3390/cells11091382/s1, Figure S1. KEGG pathways for ‘alpha’ comparisons. (A) MicroRNA in cancer. (B) Pathways in cancer. (C) Leukocyte transendothelial migration. (D) Signaling pathways regulating pluripotency of stem cells. (E) Cell adhesion molecules. (F) Wnt signaling pathway. (G) Axon guidance. (H) MAPK signaling pathway. (I) Legend. Figure S2. KEGG pathways for ‘gamma’ comparisons. (A) MicroRNA in cancer. (B) Pathways in cancer. (C) Ras signaling pathway. (D) PI3K-Akt signaling pathway. (E) Amoebiasis. (F) ECM-Receptor interaction. (G) Focal adhesion. (H) Protein digestion and absorption. (I) Axon guidance. (J) MAPK signaling pathway. (K) Legend. Figure S3. Networks containing top genes for all comparisons. (A) WK/KK. (B) KA/KK. (C) KC/KK. (D) WA/KK. (E) WC/KK. (F) Legend with investigated processes.
