# Supplementary figures and images for: Determination of WWOX Function in Modulating Cellular Pathways Activated by AP-2α and AP-2γ Transcription Factors in Bladder Cancer
Source: Cells. 2022 Apr 19;11(9):1382. doi: 10.3390/cells11091382 (PMC9106060; doi:10.3390/cells11091382)

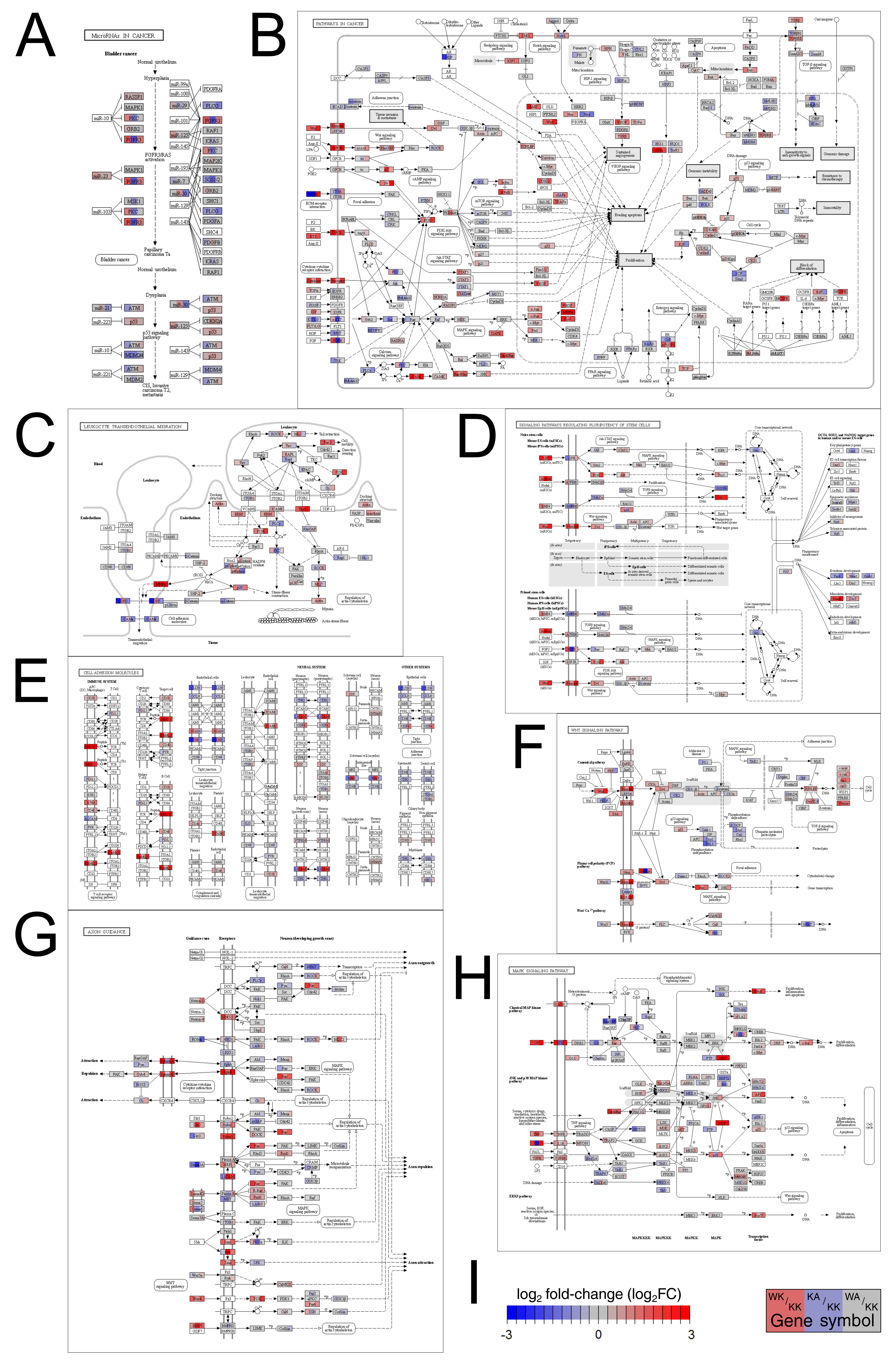

Supplement: Supplementary file 1 [file cells-11-01382-s001.zip › Figure_S1.tif]

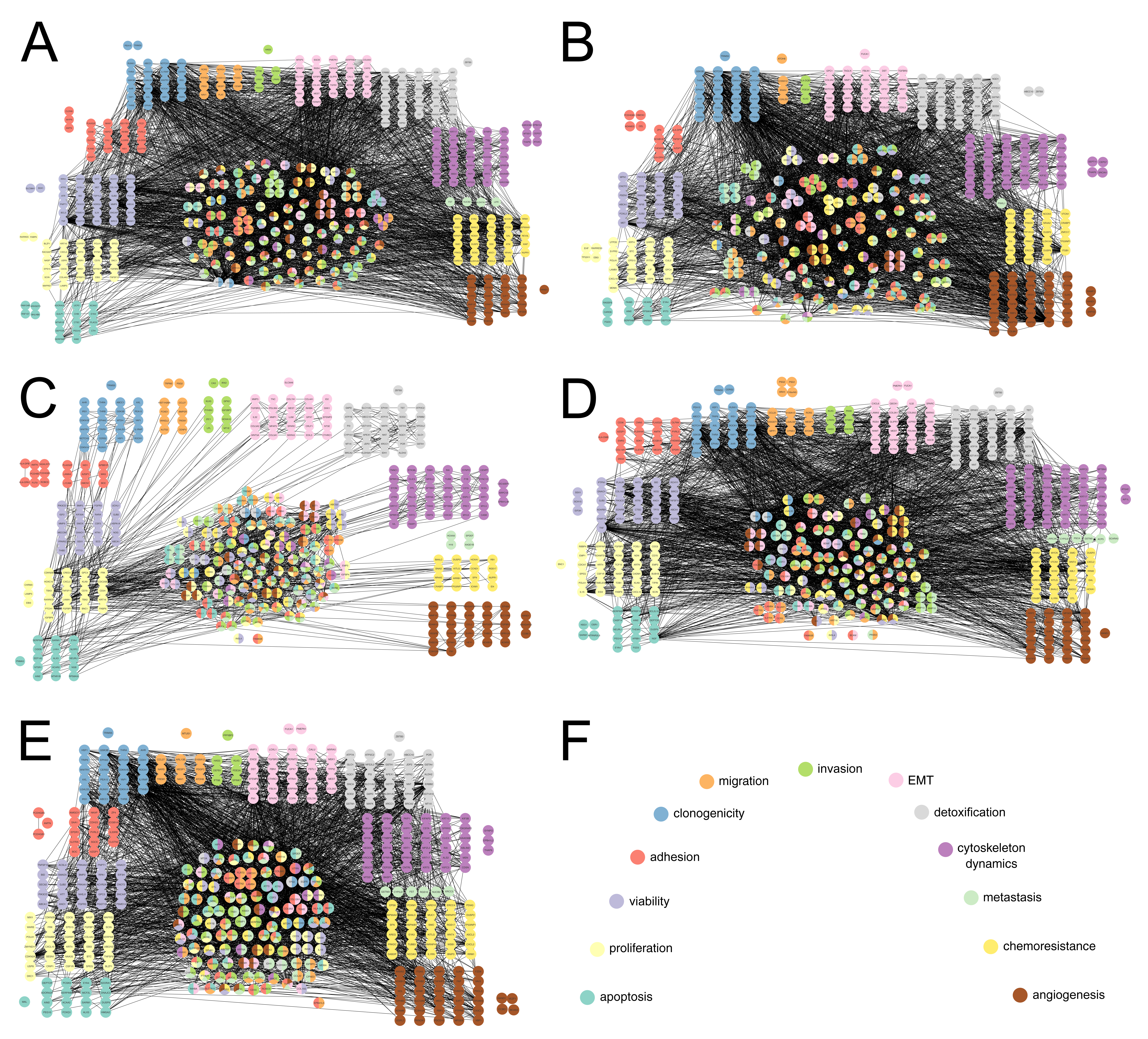

Supplement: Supplementary file 1 [file cells-11-01382-s001.zip › Figure_S3.tif]
